# Developing a Warning Model of Potentially Inappropriate Medications in Older Chinese Outpatients in Tertiary Hospitals: A Machine-Learning Study

**DOI:** 10.3390/jcm12072619

**Published:** 2023-03-30

**Authors:** Qiaozhi Hu, Fangyuan Tian, Zhaohui Jin, Gongchao Lin, Fei Teng, Ting Xu

**Affiliations:** 1 Department of Pharmacy, West China Hospital, Sichuan University, Chengdu 610041, China; 2 School of Information Science and Technology, Southwest Jiaotong University, Chengdu 611756, China

**Keywords:** older, outpatient, potentially inappropriate medications, machine learning

## Abstract

Due to multiple comorbid illnesses, polypharmacy, and age-related changes in pharmacokinetics and pharmacodynamics in older adults, the prevalence of potentially inappropriate medications (PIMs) is high, which affects the quality of life of older adults. Building an effective warning model is necessary for the early identification of PIMs to prevent harm caused by medication in geriatric patients. The purpose of this study was to develop a machine learning-based model for the warning of PIMs in older Chinese outpatients. This retrospective study was conducted among geriatric outpatients in nine tertiary hospitals in Chengdu from January 2018 to December 2018. The Beers criteria 2019 were used to assess PIMs in geriatric outpatients. Three problem transformation methods were used to tackle the multilabel classification problem in prescriptions. After the division of patient prescriptions into the training and test sets (8:2), we adopted six widely used classification algorithms to conduct the classification task and assessed the discriminative performance by the accuracy, precision, recall, F1 scores, subset accuracy (ss Acc), and Hamming loss (hm) of each model. The results showed that among 11,741 older patient prescriptions, 5816 PIMs were identified in 4038 (34.39%) patient prescriptions. A total of 41 types of PIMs were identified in these prescriptions. The three-problem transformation methods included label power set (LP), classifier chains (CC), and binary relevance (BR). Six classification algorithms were used to establish the warning models, including Random Forest (RF), Light Gradient Boosting Machine (LightGBM), eXtreme Gradient Boosting (XGBoost), CatBoost, Deep Forest (DF), and TabNet. The CC + CatBoost model had the highest accuracy value (97.83%), recall value (89.34%), F1 value (90.69%), and ss Acc value (97.79%) with a good precision value (92.18%) and the lowest hm value (0.0006). Therefore, the CC + CatBoost model was selected to predict the occurrence of PIM in geriatric Chinese patients. This study’s novelty establishes a warning model for PIMs in geriatric patients by using machine learning. With the popularity of electronic patient record systems, sophisticated computer algorithms can be implemented at the bedside to improve medication use safety in geriatric patients in the future.

## 1. Introduction

The problem of population aging has become increasingly serious, and the attention of all parties to the health of older people has continued to increase [1]. China has become the country with the largest elderly population in the world. By the end of 2019, the number of people aged 65 and above was 176 million, accounting for 12.6% of the total population [2]. According to predictions, the degree of China’s population aging will reach its peak from 2035 to 2050 [2]. In 2050, the total number of people aged 65 years and older in China will reach 380 million, accounting for nearly 30% of the total population, and the geriatric population aged 80 years and older in China will reach 120 million, accounting for nearly 10% of the total population [2].

Older patients often suffer from multiple chronic noncommunicable diseases, compared to other age groups. According to a Chinese study, 42.0% of older patients suffered from two or more chronic diseases at the same time in China, among which hypertension, diabetes, coronary heart disease, stroke, and chronic respiratory diseases were more common [3]. Multiple diseases can lead to difficulty in treatment and increased drug use [4]. As the complexity of pharmacotherapy has increased with increasing medication use, the safe use of medication has become an increasingly important area of research in older adults [5].

Potentially inappropriate medication (PIM) is a term used to describe the use of a medicine for which the associated risks outweigh the potential benefits, especially when more effective alternatives are available [6,7]. Since older patients often deal with age-related pharmacokinetic and pharmacodynamic changes, they are with high prevalence rates of PIMs (from 18 to 40%) [7,8,9,10]. In this population, PIM use can result in decreased efficacy [11] and increased avoidable adverse drug events [12], including falls, fractures, delirium, and increased mortality [13,14,15]. In recent years, many strategies and tools have been developed to assess the appropriateness of medication use in older people. Among these tools, the American Geriatrics Society (AGS) criteria have been the most commonly used and is a worldwide renowned list of PIMs.

The AGS Beers Criteria lists PIMs that are typically best avoided by older adults in most circumstances or under specific situations, such as in certain diseases or conditions. The 2019 updated AGS Beers criteria included a total of 99 PIMs, which could be divided into six categories [16]. The AGS Beers criteria contained a large number of PIMs, and hence evaluators should spend much time performing manual evaluations. In addition, the difference in the degree of familiarity with the AGS Beers Criteria could lead to the high heterogeneity of evaluation results by different institutions or evaluators. Therefore, it is necessary to implement computer algorithms to quickly and accurately identify PIMs to simplify the manual evaluation process and reduce heterogeneity.

Machine learning has been widely used in medical fields, including disease prediction or warning [17]. Machine learning has great advantages when dealing with massive data with both high-dimensional attributes and a tremendous number of instances, which is hard to conduct by conventional regression models [18]. In addition, the information on prescriptions involved multilabel classification problems. Therefore, problem transformation methods should be used to map the multilabel learning task into one or more single-label learning tasks, which resulted in improved model performance [19]. In this study, we aim to apply problem transformation models to identify correlations in prescription information and attempt to use several machine learning algorithms to find an optimal model for the warning of PIMs in geriatric outpatients.

## 2. Materials and Methods

### 2.1. Study Setting and the Study Population

The data of this study come from our previous study [20]. This retrospective study was conducted in hospitals in Chengdu, which had a population of 21.19 million and an area of 14,335 square kilometers in 2021. The patient prescriptions came from the “Cooperation Project of Hospital Prescription Analysis” [20]. A cluster sampling method was used to extract prescription data from nine of all tertiary hospitals in Chengdu [20]. These hospitals had complete outpatient departments and electronic information systems, which could provide high-level specialist medical and health services and perform higher education and scientific research tasks in several regions.

The prescriptions of older adults (aged 65 and older) who visited the outpatient clinics of the geriatric departments or geriatric centers between 1 January 2018 and 31 December 2018 in Chengdu were included [20]. Then, prescriptions with missing or incomplete information, including sex, diagnosis, medication, and dosage were excluded [20]. Solvent substances were not included when calculating the number of medications [20]. This study was retrospective and all data used in this study were fully anonymized. This study received ethics approval and approval for a waiver of informed consent from the Sichuan University West China Hospital Research Ethics Board (2020-651).

### 2.2. Data Collection

Demographic and clinical data from geriatric outpatient records were collected. The following data were documented: basic information (region, year, and department), demographic information (prescription number, age, sex, and diagnosis), and medication information (generic name, trade name, specification, dosage form, administration route, number of medications, dosage, and frequency of administration) [20].

### 2.3. Evaluation Criteria

Two trained researchers independently reviewed the medications prescribed and identified PIMs by using the AGS 2019 Beers criteria (Supplementary Material) [16]. If there was a disagreement, the decision was made by a third person. These criteria were divided into six categories [16]: (1) potentially inappropriate medication use in older adults, (2) potentially inappropriate medication use in older adults due to drug-disease or drug-syndrome interactions, (3) drugs to be used with caution in older adults, (4) potentially clinically important drug–drug interactions, (5) medications that should be avoided or the dosage of which should be reduced with varying levels of kidney function in older adults, and (6) drugs with strong anticholinergic properties.

### 2.4. Multilabel Classification

The identification of PIMs in the elderly needed several factors, including basic information (gender, age), medication, and disease. Taking these factors as independent variable $\;x_{i}$, we could get the feature set $\;X=\left\{x_{1},x_{2},\ldots ,x_{n}\right\}$. The dependent variable was the PIM set $\;y=\left\{y_{1},y_{2},\ldots ,y_{m}\right\}$, where $\;y_{i}\in \left\{0,1\right\}$ indicated whether the prescription was with a certain PIM.

**Figure 1:**
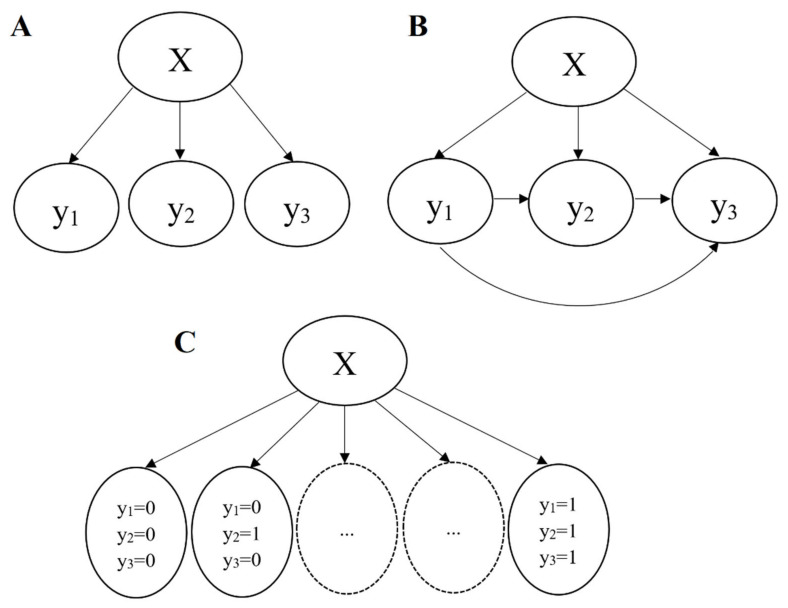
Transformation methods of multilabel classification problems, X represents the data features, $\;y_{i}$ represents the calculation results for the $i^{th}$ label. (**A**) Binary relevance (BR) transforms the multilabel data with 3 labels into 3 independent binary datasets. (**B**) The transformation of the multilabel dataset by classifier chains CC. (**C**) The process of label power set (LP) for multilabel data.

Three problem transformation methods were used to tackle the multilabel classification problem by transforming it into other well-established learning problems, including binary relevance (BR), classifier chains (CC), and label power set (LP) [21]. The modes of BR, CC, and LP are depicted in Figure 1.

The BR approach is a single-endpoint model that treats each label as an independent binary problem but does not take into account the dependencies between the labels [22,23]. So, the BR approach is often used as a baseline reference. In the CC approach, the labels and their corresponding classifiers are chained, such that subsequent classifiers in the chain can learn and relate their target label to the prior labels in the chain [24]. The CC approach can overcome the disadvantage of not considering dependencies between labels and capture possible dependencies between the labels [25]. The LP approach can transform the multilabel classification problem into a single-label multiclass problem, resulting in a single classifier which treats each unique label vector as a class [26]. Therefore, the LP approach may be infeasible for problems with many labels due to the exponential growth of the number of classes relative to the number of labels.

### 2.5. Model Development

Prescriptions were randomly stratified (8:2) into the training set to develop models and the test set to evaluate the performance of the models. Then, six widely used classification algorithms were adopted to conduct the classification task to find the best warning models with our dataset, including Random Forest (RF), Light Gradient Boosting Machine (LightGBM), eXtreme Gradient Boosting (XGBoost), CatBoost, Deep Forest (DF), and TabNet. Data were analyzed by using Python software (version 3.8) (Python Software Foundation, Reston, VA, USA).

### 2.6. Model Evaluation Metrics

We used various evaluation measures to evaluate and compare models, including accuracy, precision, recall, F1 scores, subset accuracy (ss Acc) and hamming loss (hm) [27,28]. The formulas are as follows:

$$\mathrm{Accuracy}=\frac{1}{N}\overset{N}{\underset{i=1}{\sum }}\left|\frac{h\left(xi\right)\cap yi}{h\left(xi\right)\cup yi}\right|$$

$$\mathrm{Precision}=\frac{1}{N}\overset{N}{\underset{i=1}{\sum }}\left|\frac{h\left(xi\right)\cap yi}{yi}\right|$$

$$\mathrm{Recall}=\frac{1}{N}\overset{N}{\underset{i=1}{\sum }}\left|\frac{h\left(xi\right)\cap yi}{h\left(xi\right)}\right|$$

$$F1=\frac{1}{N}\overset{N}{\underset{i=1}{\sum }}\frac{2\times \left|h\left(xi\right)\cap yi\right|}{\left|h\left(xi\right)\right|+\left|yi\right|}$$

$${\mathrm{Accuracy}}_{sub}=\frac{1}{N}\overset{N}{\underset{i=1}{\sum }}\left|h\left(xi\right)=yi\right|$$

$$\mathrm{Hamming}\;\mathrm{loss}=\frac{1}{N}\overset{N}{\underset{i=1}{\sum }}\frac{1}{Q}\left|h\left(xi\right)\Delta yi\right|$$

where $y_{i}$ denotes the set of true labels of example $\;x_{i}$; $h(x_{i})$ denotes the set of predicted labels for the same sample; Δ stands for the symmetric difference between the two sets; N is the number of examples; and Q is the total number of possible class labels [27,28]. F1 is used to measure the pros and cons of the model, and the larger the value is, the better the model performance [27,28]. The subset accuracy means the proportion of predicted and true label sets that are exactly the same, which is the strictest evaluation metric since a multiclass example is considered correctly classified if and only if all the labels in the example are correctly classified for subset accuracy [27,28]. The Hamming loss is used to measure the proportion of labels predicted incorrectly in the entire test set [27,28]. The lower the value is, the higher the performance of the classifier, as this is a loss function [27,28].

### 2.7. Statistical Analysis

Categorical variables were summarized using frequency counts and percentages, and continuous variables were presented as the means with standard deviations (SD) and medians with ranges. Comparisons between groups were made using the nonparametric Mann–Whitney U test for continuous variables and the χ^2^ test for categorical variables. These analyses were conducted using SPSS 25.0 software (IBM Information Management, Chicago, IL, USA).

## 3. Results

### 3.1. Study Population

A total of 50,492 patient prescriptions were registered during the study period. The following prescriptions were excluded: 9140 were age inconsistencies, 542 were incomplete diagnoses, 298 were blank diagnoses, 4 missed genders, and 29 were only solvent prescriptions. After randomly selecting nine hospitals in Chengdu, a total of 11,741 patient prescriptions were enrolled in this study.

Among the patient prescriptions enrolled, the mean age was 78.69 ± 8.29 years (range 65 to 119 years), and females represented 40.12% (4711/11,741). The median number of diseases per patient was two (range 1 to 19), and 18.11% (2126/11,741) were prescribed to patients who suffered from at least five diseases. The median number of medications per patient was three (range 1 to 23). The enrolled patients were divided into training and testing sets at a ratio of 8:2, with 9263 and 2349 patients, respectively. There were no significant differences in any variables between the training and testing sets (*p* > 0.05) (Table 1).

Potentially inappropriate medication

**Figure 2:**
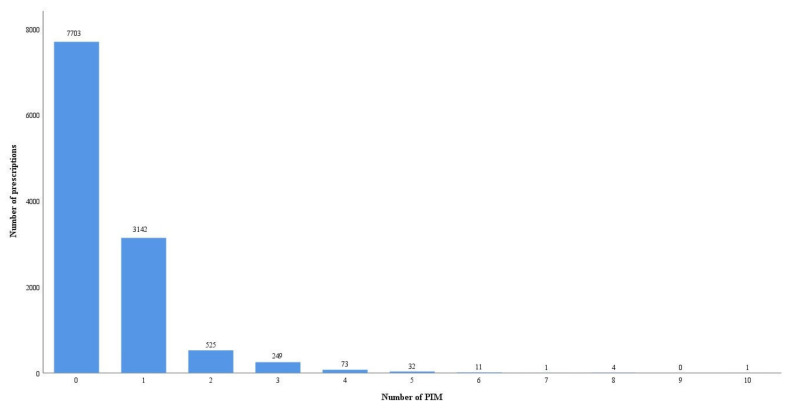
The number of PIMs and prescriptions.

**Figure 3:**
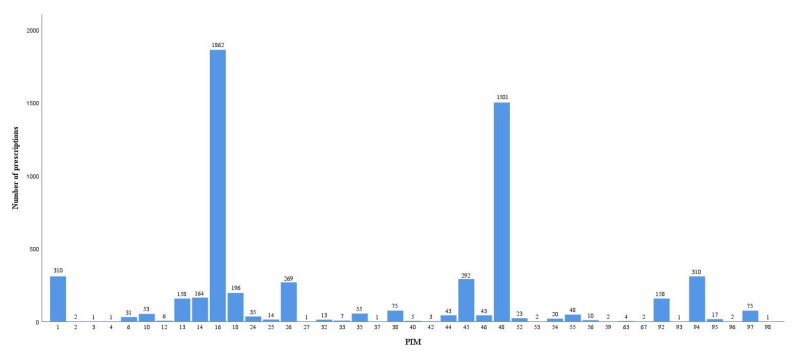
The number of prescriptions in each PIM.

A total of 4038 (34.39%) patient prescriptions were with PIMs based on the pharmacists’ review. Among these prescriptions with PIMs, 896 patient prescriptions had more than one PIM, and a total of 5816 PIMs were identified, as shown in Figure 2. Based on the AGS 2019 Beers criteria [16], 41 types of PIMs were identified in these prescriptions. The common PIMs in Chinese geriatric inpatients were No. 16 (avoiding benzodiazepines; N = 1862; 32.02%), No. 48 (using antipsychotics, diuretics, tramadol, or some types of antidepressants; N = 1501; 25.81%), No. 1 and No. 94 (avoiding anticholinergics; N = 310; 5.33%), and No. 45 (using aspirin for primary prevention of cardiovascular disease and colorectal cancer with caution in adults ≥70 years; N = 292; 0.5%), as shown in Figure 3.

### 3.2. Model Performance

According to the AGS 2019 Beers criteria [16], the data for analysis included the independent variable (gender, age, medication, and disease) and the dependent variable (type of PIM). Among the independent variable, 526 medications and 2257 diseases were identified. Among the dependent variable, 41 types of PIMs were identified.

The comparison of the six classification models in different problem transformation methods is shown in Table 2. Using classifier chains as the multilabel classification model, CatBoost outperformed the other models. The CC + CatBoost model had the highest accuracy value (97.83%), recall value (89.34%), F1 value (90.69%), and ss Acc value (97.79%), with a good precision value (92.18%) and the lowest hm value (0.0006). Therefore, the CC + CatBoost model was selected to warn of the occurrence of PIMs in geriatric Chinese patients.

The results of the evaluation of each PIM in the test set by the CC + CatBoost model are shown in Table 3.

## 4. Discussion

Drug-related problems are prevalent in the older adult population and pose a major patient safety concern [29]. Developing AGS Beers Criteria to identify potentially inappropriate medication, which is closely associated with adverse clinical outcomes, is to improve medication selection, educate clinicians and patients, and reduce adverse drug events [16]. Meanwhile, it can also serve as a tool for evaluating the quality of care, cost, and patterns of drug use of older adults [16]. Since the evaluations are time consuming and the results of the evaluation often have large differences by different evaluators [30], the use of the criteria is limited. Several clinical decision support systems (CDSSs) have been used to improve appropriate prescribing in this population in some countries [31,32,33]. These CDSSs identified PIMs based on the keywords of the established database such that the identification of PIM by these systems might be less accurate facing unknown independent variables (diseases or medications).

In this study, we established a novel warning model for PIMs in geriatric patients by using machine learning. Machine learning algorithms have been utilized in a variety of medical applications in the twenty-first century, including providing supportive information or additional aids for improving the accuracy and efficiency of diagnosis and treatment [33] and developing models to predict prognosis [34,35]. Machine learning with faster data processing and improved computer functions can process a large amount of data in a short time, leading to rapid advances [36]. It can process complex nonlinear relationships between variables and outcomes and has the ability to learn from data situations in the real world [36]. Therefore, it can identify PIMs more accurately when prescriptions have unknown independent variables. In addition, traditional classification algorithms consider learning problems that contain only one label, i.e., each example is associated with one single nominal target variable characterizing its property [19]. Due to the presence of multiple target variables in prescriptions, problem transformation methods should be used to transform the multilabel classification problem into several single-label classification problems. In this study, we attempted to apply three problem transformation methods to tackle the multilabel classification problem and six classification algorithms to establish warning models. These machine learning methods have good performances, especially Classifier Chain + Catboost. The precision of the CC + Catboost model was satisfactory, with the highest accuracy value, recall value, F1 value, ss Acc value, and the lowest hm value. Therefore, the CC + CatBoost model was selected to warn of the occurrence of PIMs in geriatric Chinese patients.

In this study, the results showed that some PIMs were rarely or even not identified in older Chinese adults, which made it difficult to evaluate the performance of the CC + Catboost model in these PIMs. A total of 58 PIMs were not identified in this study population, including all fifth-category PIMs. The fifth category has 28 PIMs, which should be identified based on the results of kidney function tests. Due to a lack of indicators of renal function in the prescriptions, we could not evaluate these PIMs. In addition, because some medicines are not commonly used or have not been approved in China, some PIMs could not also be found in this study, such as nitrofurantoin, reserpine, disopyramide, and dronedarone. For a similar reason, some PIMs were rare in older Chinese adults and were only identified in one to five patient prescriptions.

A total of 11,741 older patient prescriptions were included, and 5816 PIMs were identified in 4038 patient prescriptions. There were 41 types of PIMs identified in these prescriptions. Among these PIMs, avoiding benzodiazepines was the most common PIM in older Chinese patients. The high rate and long-term use of benzodiazepines can be attributed to the high prevalence of insomnia in geriatric patients. The Canadian Study on Health and Aging (2000) reported that the risk of long-term use of benzodiazepines in the older adult population was 5.5 times higher than in the younger adult population [37], and the rate of the utilization of benzodiazepines in older adults continued to increase [38]. However, the long-term use of benzodiazepines does not appear to work well to treat sleep disturbances [39]. In general, benzodiazepines can increase the risk of cognitive impairment, delirium, falls, fractures, and motor vehicle crashes in older adults [16,38,40]. Benzodiazepine misuse is also associated with past-year suicidal ideation in older adults [41]. For older adults with chronic insomnia, cognitive behavioral therapy and benzodiazepine receptor agonists are the recommended treatments [16,42,43]. Currently, due to a large number of older patients and the shortage of medical resources in China, it may be difficult for these recommended treatments to be applied on a large scale among Chinese older adults at present.

Since antipsychotics, diuretics, tramadol, and some types of antidepressants may exacerbate or cause a syndrome of inappropriate antidiuretic hormone or hyponatremia, the use of these medicines in older patients is considered PIM [16], which is also most common in Chinese geriatric patients. Among these medicines, diuretics, and SSRIs were the most commonly used in this study. Due to eliminating edema and maintaining stable blood flow, diuretics are commonly used drugs for patients with cardiovascular diseases. Diuretic-related hyponatremia is a prevalent cause for admission, especially hydrochlorothiazide and indapamide [44]. SSRIs, including citalopram, escitalopram, fluoxetine, paroxetine, and sertraline, were highly prevalent in this study population. Several studies have reported hyponatremia associated with SSRI use, with the incidence varying from 0.5% to 32% [45,46,47,48]. Older age served as a risk factor for the development of hyponatremia with SSRIs [49]. Due to the adverse outcomes associated with hyponatremia (i.e., impaired cognition, falls and fractures, and mortality), recognition of drug-induced hyponatremia is of vital importance, while responsible agents should be discontinued, and “rechallenge” should be avoided by informing the patient and involved caregivers.

There are several limitations to consider. First, the Beers Criteria were developed by the American Geriatrics Society Beers Criteria Update Expert Panel. Although the AGS Beers Criteria are widely used worldwide, some PIMs mentioned in it are rare or even absent in China. The reason for this was that some medicines or medical tests were not commonly used or approved in China. Second, although the total number of older patient prescriptions enrolled was 11,741, cases of some PIMs were still lacking, which caused the performance of the CC + Catboost model in these PIMs to not be evaluated. For the unusual PIMs in China, sufficient prescriptions should be collected to improve machine learning performance in future research. Furthermore, the patient’s personal information and out-of-hospital data cannot be obtained, such as biochemical tests, weight, smoking history, medication history, and history of adverse drug reactions, which can lead to the detection rate of PIM prescriptions being underestimated or overestimated.

## 5. Conclusions

This is a novel study to establish a warning model for potentially inappropriate medications in elderly Chinese patients by using machine learning. In this study, a total of 41 types of PIMs were identified in 11,741 patient prescriptions. Three problem transformation methods and six classification algorithms were used to develop the warning model. Among these models, Classifier Chain + Catboost outperforms the other models. Developing an appropriate warning model for potentially inappropriate medications in older outpatients could be used to quickly and accurately identify PIMs, simplify the manual evaluation process, and reduce heterogeneity between different evaluators. Therefore, we consider that determining warning models is an effective way to improve the quality performance of the evaluation of PIMs, reduce the incidence of PIMs, and prevent harm caused by medication in elderly patients. Furthermore, with the popularity of electronic patient record systems and the wide availability of structured patient data, this sophisticated computer algorithm can be implemented at the bedside to improve inpatient safety in clinical practice in the future.

## Figures and Tables

**Table 1:** Patient Characteristics. PIM: Potentially Inappropriate Medication.

Variable	Total (*n* = 11,741)	Training Set (*n* = 9392)	Testing Set (*n* = 2349)	*p*
PIM				
Yes	4038	3204	834	0.410
No	7703	6188	1515	
Gender				
Male	7030	5607	1423	0.981
Female	4711	3785	926	
Age (years)	78.69 ± 8.29 (65–119)	78.71 ± 8.29 (65–103)	78.69 ± 8.29 (65–119)	0.935
Number of diseases	2.84 ± 2.73 (1–19)	2.86 ± 2.75 (1–19)	2.80 ± 2.69 (1–18)	0.335
Number of medications	3.39 ± 2.59 (1–23)	3.40 ± 2.59 (1–18)	3.40 ± 2.62 (1–23)	0.975

**Table 2:** Model Performance.

Problem Transformation Method	Classification Model	Accuracy	Precision	Recall	F1	ss Acc	hm
BR	RF	0.9332	0.7871	0.5415	0.6121	0.9293	0.0023
	LightGBM	0.9293	0.6911	0.7005	0.6778	0.9285	0.0022
	XGBoost	0.9706	0.9000	0.8367	0.8624	0.9698	0.0008
	CatBoost	0.9762	0.9098	0.8685	0.8880	0.9753	0.0007
	DF	0.9617	0.8095	0.7796	0.7876	0.9600	0.0011
	TabNet	0.8966	0.6356	0.5449	0.5719	0.8923	0.0035
CC	RF	0.9281	0.7994	0.5457	0.6208	0.9276	0.0025
	LightGBM	0.9395	0.8137	0.7856	0.7859	0.9378	0.0018
	XGBoost	0.9715	0.9228	0.8712	0.8933	0.9706	0.0008
	CatBoost	0.9783	0.9218	0.8934	0.9069	0.9779	0.0006
	DF	0.9621	0.8194	0.8026	0.8090	0.9608	0.0011
	TabNet	0.8859	0.7043	0.5592	0.5939	0.8765	0.0039
LP	RF	0.9204	0.7903	0.5491	0.6309	0.9195	0.0031
	LightGBM	0.7220	0.3219	0.3779	0.3109	0.7088	0.0209
	XGBoost	0.9434	0.8523	0.5646	0.6252	0.9425	0.0020
	CatBoost	0.9447	0.7601	0.6224	0.6764	0.9442	0.0020
	DF	0.9421	0.7683	0.6556	0.7013	0.9413	0.0021
	TabNet	0.8889	0.5762	0.4474	0.4618	0.8710	0.0067

BR: Binary Relevance; CC: Classifier Chain; LP: Label Power Set; RF: Random Forest; LightGBM: Light Gradient Boosting Machine; XGBoost: eXtreme Gradient Boosting; DF: Deep Forest; ss Acc: subset accuracy; hm: hamming loss.

**Table 3:** The performance of each PIM by the CC + CatBoost model in the test set. PIM: Potentially Inappropriate Medication.

No of PIM	Sample, *n*	Precision	Recall	F1
1	63	1.0000	0.9841	0.9920
2	0	0.0000	0.0000	0.0000
3	0	0.0000	0.0000	0.0000
4	0	0.0000	0.0000	0.0000
6	7	1.0000	0.8571	0.9231
10	9	1.0000	0.8889	0.9412
12	1	0.0000	0.0000	0.0000
13	33	1.0000	1.0000	1.0000
14	36	1.0000	1.0000	1.0000
16	358	1.0000	1.0000	1.0000
18	43	1.0000	1.0000	1.0000
24	9	1.0000	0.5556	0.7143
25	3	1.0000	1.0000	1.0000
26	61	1.0000	1.0000	1.0000
27	1	0.0000	0.0000	0.0000
32	3	1.0000	1.0000	1.0000
33	0	0.0000	0.0000	0.0000
35	9	1.0000	0.5556	0.7143
37	1	0.0000	0.0000	0.0000
38	17	1.0000	0.5882	0.7407
40	0	0.0000	0.0000	0.0000
42	0	0.0000	0.0000	0.0000
44	3	1.0000	0.6667	0.8000
45	65	0.8772	0.7692	0.8197
46	9	0.8182	1.0000	0.9000
48	299	1.0000	1.0000	1.0000
52	3	1.0000	1.0000	1.0000
53	1	0.0000	0.0000	0.0000
54	6	1.0000	1.0000	1.0000
55	11	1.0000	0.8182	0.9000
56	2	0.0000	0.0000	0.0000
59	0	0.0000	0.0000	0.0000
64	1	1.0000	1.0000	1.0000
67	1	0.0000	0.0000	0.0000
92	33	1.0000	1.0000	1.0000
93	0	0.0000	0.0000	0.0000
94	63	1.0000	0.9841	0.9920
95	3	1.0000	1.0000	1.0000
96	0	0.0000	0.0000	0.0000
97	15	1.0000	1.0000	1.0000
98	0	0.0000	0.0000	0.0000

## Data Availability

The data presented in this study are available in the present manuscript.

## Supplementary Materials

The following supporting information can be downloaded at: https://www.mdpi.com/article/10.3390/jcm12072619/s1.
